# Genotype–Phenotype Variability in Congenital Thrombotic Thrombocytopenic Purpura (TTP): Two Novel ADAMTS13 Variants

**DOI:** 10.7759/cureus.97714

**Published:** 2025-11-24

**Authors:** Nita Radhakrishnan, Archit S Pandharipande, Hari Gaire, Savitri Singh

**Affiliations:** 1 Department of Pediatric Hematology Oncology, Post Graduate Institute of Child Health, Noida, IND; 2 Department of Pathology, Post Graduate Institute of Child Health, Noida, IND

**Keywords:** congenital thrombotic thrombocytopenic purpura, genotype–phenotype variability, novel adamts13 variants, pediatric cttp, recombinant adamts13

## Abstract

Congenital thrombotic thrombocytopenic purpura (cTTP; Upshaw-Schulman syndrome) is an ultra-rare hereditary thrombotic microangiopathy caused by biallelic pathogenic variants in ADAMTS13. The condition is characterized by microangiopathic hemolytic anemia, thrombocytopenia, and ischemic organ injury. Although more than 450 variants have been described, genotype-phenotype correlations remain imperfect, and presentation is highly heterogeneous. We describe two children with cTTP, illustrating opposite ends of the clinical spectrum.

Case 1: a 10-year-old girl with recurrent anemia and thrombocytopenia since early childhood was found to have severe ADAMTS13 deficiency (<3%) without inhibitor. Genetic testing identified a novel homozygous missense variant c.946G>C (p.Gly316Arg) in the cysteine-rich domain, absent from population databases and classified as likely pathogenic. She remains stable on prophylactic fresh frozen plasma infusions every 3-4 weeks, though complicated by allergic reactions and progressive renal dysfunction.

Case 2: an 11-month-old infant, born to consanguineous parents with a history of sibling deaths in infancy, presented with transfusion-dependent anemia, thrombocytopenia, and subsequently an ischemic stroke. Exome sequencing revealed a novel homozygous missense variant c.1519C>T (p.Arg507Trp) in the spacer domain, also classified as likely pathogenic. Despite repeated plasma infusions, the child suffered progressive neurological deterioration and succumbed in infancy.

These cases highlight the wide clinical heterogeneity of cTTP. While N-terminal variants are often associated with early severe disease, and distal domain variants with later onset, residual activity alone does not predict outcome. Multiparametric pathogenicity assessment (SIFT, PolyPhen, conservation, charge, phosphorylation, codon usage) combined with ACMG criteria is essential to classify novel variants. Plasma infusion remains the mainstay of therapy, but recombinant ADAMTS13 holds promise to transform care and reduce treatment burden. cTTP should be considered in any child presenting with anemia, thrombocytopenia, and schistocytosis. Early biochemical and genetic confirmation enables timely plasma therapy and counseling. Novel ADAMTS13 variants continue to expand the mutational spectrum and underscore the variability of genotype-phenotype correlations.

## Introduction

Congenital thrombotic thrombocytopenic purpura (cTTP; Upshaw-Schulman syndrome) is a rare hereditary thrombotic microangiopathy characterized by severe deficiency of ADAMTS13 activity. The clinical spectrum ranges from severe neonatal jaundice and thrombocytopenia to relapsing or chronic microangiopathic hemolysis with ischemic organ injury in childhood or adulthood. Registry data suggest high morbidity and mortality, particularly when diagnosis is delayed [1]. More than 400 ADAMTS13 variants have been reported, distributed across functional domains [2]. Although trends between genotype and phenotype are recognized, clinical heterogeneity remains substantial. Here, we report two pediatric cases with novel pathogenic mutations causing cTTP, illustrating extremes of presentation, and contextualize them with available pediatric literature, highlighting challenges in pathogenicity assessment and treatment advances.

## Case presentation

Case one

**Figure 1:**
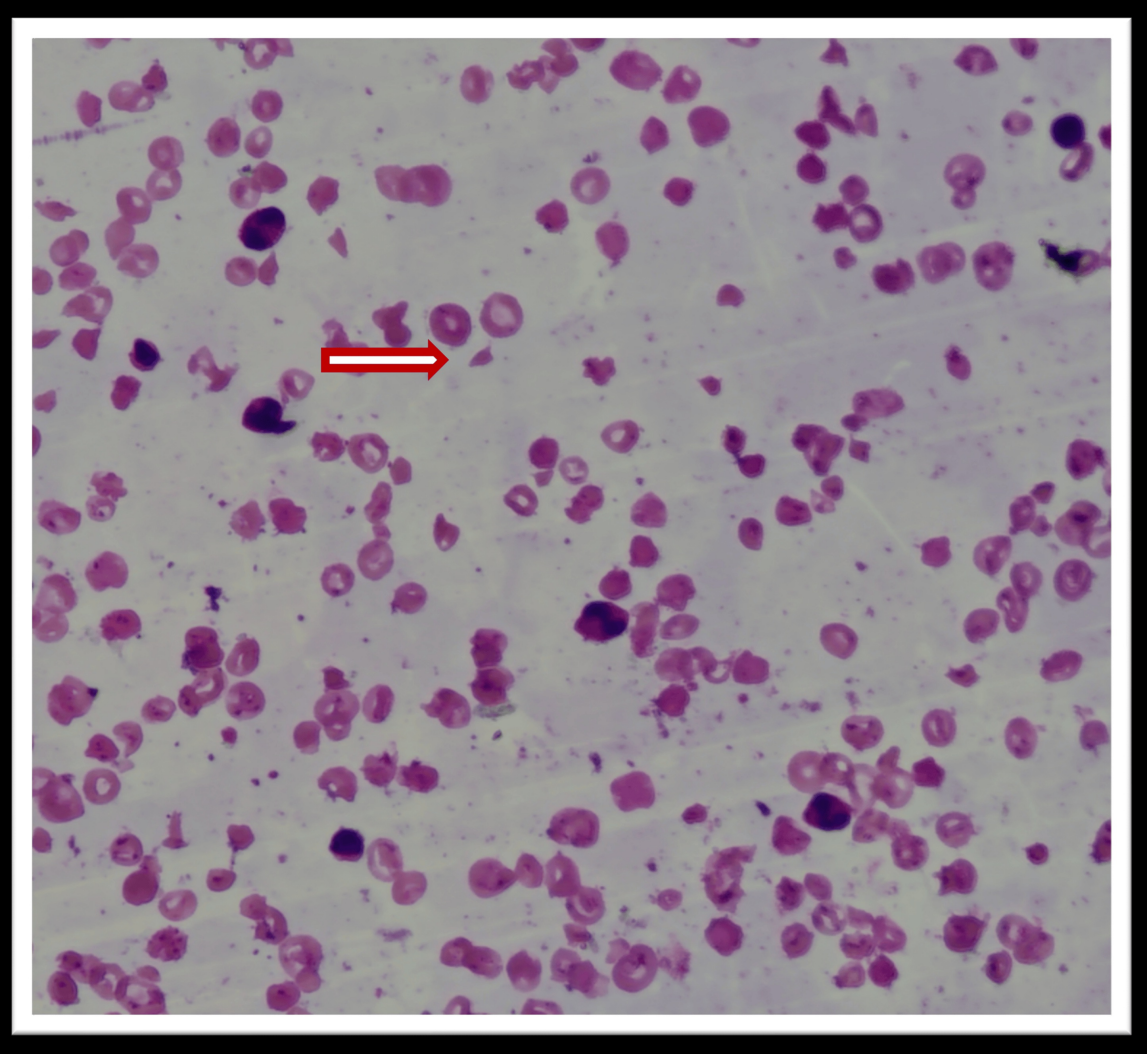
Red cell fragmentation was noted on the peripheral smear of case one

A 10-year-old girl, born to non-consanguineous parents, presented with progressive pallor, abdominal pain, ecchymotic patches, and vomiting. Her birth history was significant for neonatal jaundice requiring phototherapy. Past medical history included diarrheal illness at 1.5 years, dehydration following sun exposure at five years, and recurrent lower respiratory tract infections. She received two transfusions of packed red blood cells prior to her diagnosis. Family history revealed a bad maternal obstetric history (two stillbirths, one first-trimester abortion) with a negative workup for antiphospholipid antibodies and lupus. On examination, she had pallor and palatal hemorrhages but no icterus, hepatosplenomegaly, or lymphadenopathy. Hematological evaluation revealed bicytopenia (anemia and thrombocytopenia). Peripheral smear showed anisopoikilocytosis, reticulocytosis, and schistocytes with a schistocyte index of 4.6%. Red cell fragmentation on peripheral smear is demonstrated in Figure 1.

Laboratory findings included elevated lactate dehydrogenase, markedly reduced serum haptoglobin, and a negative direct antiglobulin test. ADAMTS13 activity was consistently <3% (reference 68-163%) with no detectable inhibitor. ANA was weakly positive, and dsDNA was negative. All investigations are detailed in Table 1.

**Table 1:** Comparison of laboratory reports of both cases of cTTP

Parameter	Normal pediatric reference	Case 1	Case 2
Hemoglobin	11.5–15.5 g/dL	7.8 g/dL during relapse	5.4 g/dL at stroke episode
Platelet count	150–400 ×10^9/L	38 ×10^9/L	11 ×10^9/L
Total leukocyte count	4–11 ×10^9/L	5×10^9/L	15 ×10^9/L (leukocytosis)
Red cell distribution width (RDW)	11.5-14.5%	18.3%	42%
Corrected reticulocyte count	0.5–1.5%	9%	4.2%
Schistocyte index	<1%	4.6%	4%
LDH	<250 U/L	499 U/L	420 U/L
Haptoglobin	30-200mg/dl	<30 mg/dl	Not done
Direct Coombs test	Negative	Negative	Negative
Bone marrow cellularity	Age-appropriate	Hypercellular	Hypercellular
Erythroid series	Normoblastic	Hyperplasia	Hyperplasia
ADAMTS13 activity	68–163%	<3%	<5%
ADAMTS13 inhibitor	Not detected	<0.4 BEU	Not detected
Renal function (Creatinine)	0.3– 0.9 mg/dL	1.8 mg/dl	0.4 mg/dl
Serum Bilirubin	Total <1.2 mg/dL	Neonatal hyperbilirubinemia	Neonatal hyperbilirubinemia
Autoimmune markers	Negative	ANA 1+ / dsDNA negative	ANA/dsDNA negative
Genetic analysis	Not applicable	c.946G>C (p.Gly316Arg)	c.1519C>T(p.Arg507Trp)
Birth history	NA	Neonatal jaundice	Neonatal jaundice
Family history	NA	Maternal bad obstetric history	2 siblings died neonatally
Clinical course	NA	Relapsing MAHA	Catastrophic infantile course

Molecular analysis identified a homozygous missense variant c.946G>C (p.Gly316Arg) in exon 8, located in the cysteine-rich domain. This variant was absent from population databases and predicted to be deleterious by multiple in silico tools. According to ACMG criteria, it was classified as likely pathogenic. The patient initially responded well to fresh frozen plasma (FFP) infusions during acute relapses. She was subsequently commenced on prophylactic plasma every 3-4 weeks. She has an allergic reaction to plasma transfusions and needs a prophylactic pheniramine injection for each transfusion. Despite an intercurrent episode of scrub typhus with renal dysfunction, she has remained stable on prophylaxis. She is 15 years old at present, has progressive renal dysfunction, and is being evaluated for renal transplantation.

Case two

**Figure 2:**
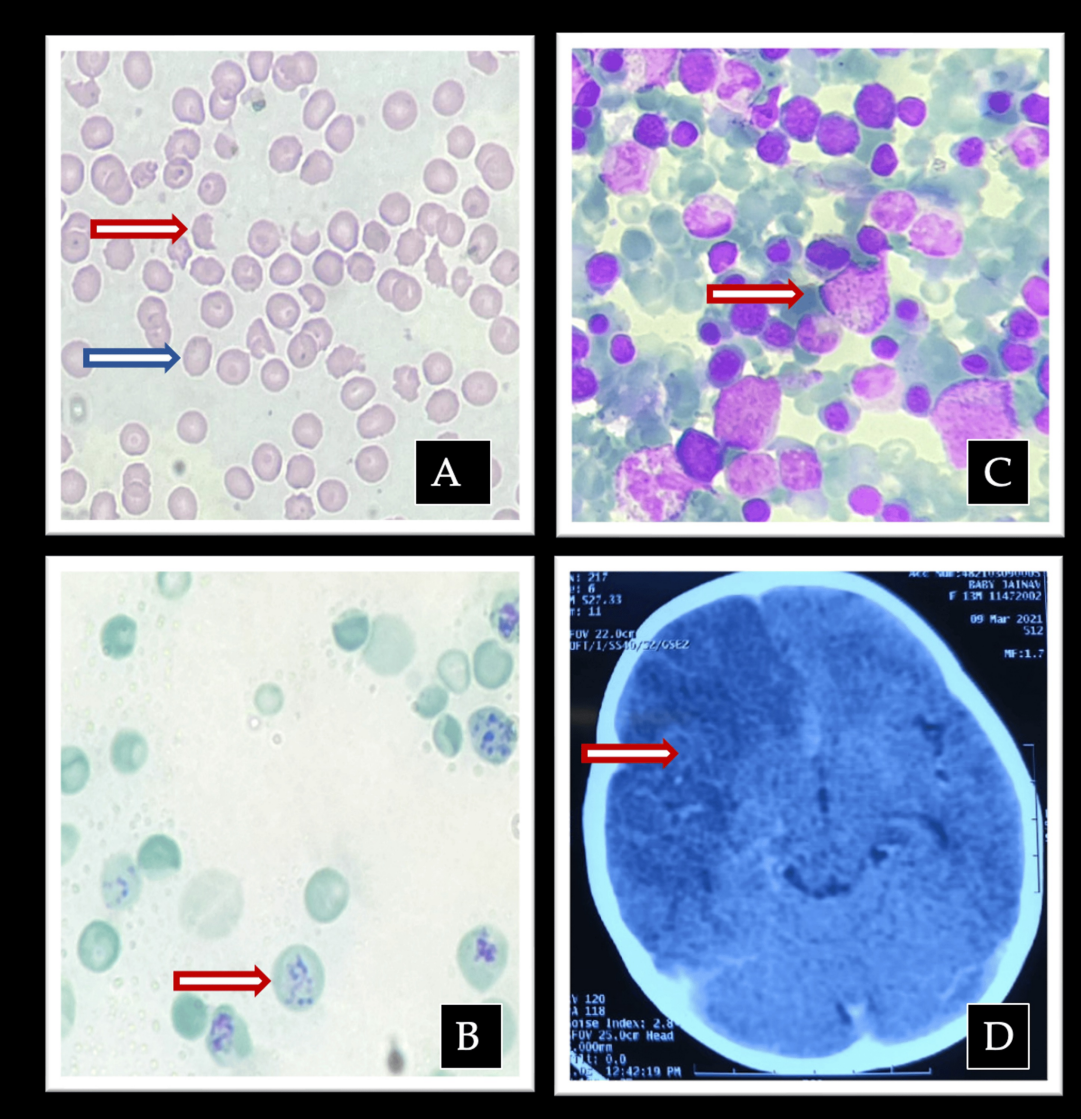
Hematopathological and neuroimaging findings of Case 2 2A: Peripheral blood smear showing schistocytes (fragmented red blood cells) and polychromasia, consistent with microangiopathic hemolytic anemia. The red arrow marks a fragmented red cell (schistocyte), and the blue arrow marks polychromasia. 2B: Reticulocyte preparation demonstrating increased reticulocytosis, reflecting marrow response to hemolysis. The arrow marks a reticulocyte. 2C: Bone marrow aspirate smear revealing reactive erythroid hyperplasia without blasts. An arrow marks an erythroid precursor cell. 2D: Computed tomography (CT) of the brain showing right middle cerebral artery infarction. The arrow marks a hypodense area in the right frontoparietal area suggestive of infarct.

An 11-month-old infant, born to consanguineous parents, presented to us with a history of pallor for the last six months. Two elder siblings had died in infancy following a similar history of pallor and transfusion requirement. She was suspected of having thalassemia major, which was ruled out by HPLC. At our center, on examination, she was noted to have pallor without any hepatosplenomegaly. A complete blood count showed hemoglobin of 3.9 g/dL with a platelet count of 4000/mL. Peripheral smear revealed marked schistocytosis, polychromasia, and reticulocytosis, with elevated lactate dehydrogenase and reduced serum haptoglobin, consistent with ongoing microangiopathic hemolysis. The direct Coombs test was negative. Bone marrow was hypercellular with erythroid hyperplasia, without blasts. Hematopathological and neuroimaging findings are demonstrated in Figure 2.

**Figure 3:**
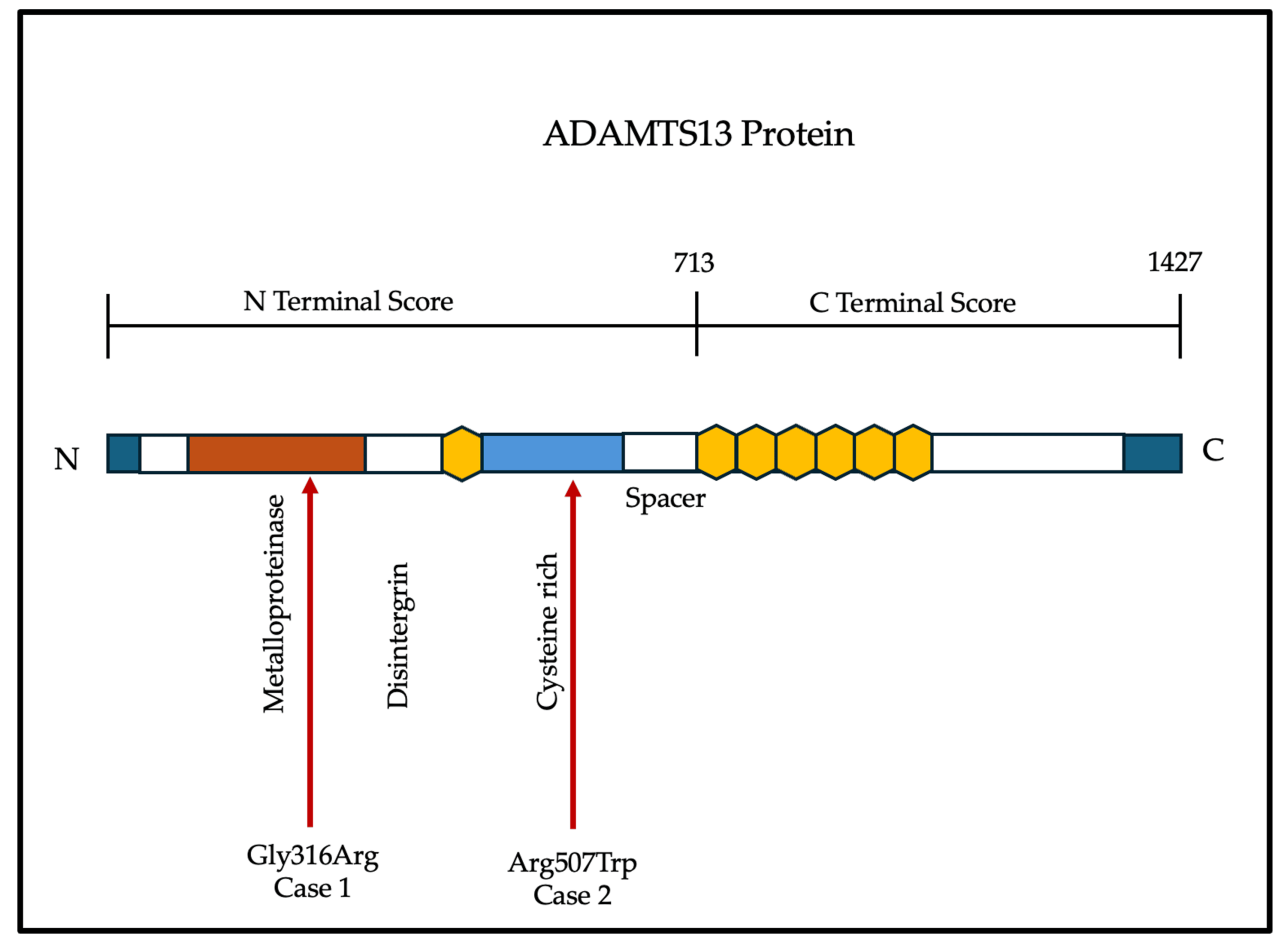
Schematic representation of the ADAMTS13 protein and location of novel variants identified in the present cases.

In view of recent transfusions, ADAMTS activity could not be sent, and instead, whole exome sequencing was advised. While on follow-up, the child presented acutely with left-sided hemiparesis and seizures. Neuroimaging demonstrated a right middle cerebral artery infarct. Exome sequencing identified a novel homozygous missense variant, c.1519C>T (p.Arg507Trp), in exon 13, located in the spacer domain. This variant was absent from population databases, conserved across species, and predicted to be damaging by multiple in silico tools (SIFT, PolyPhen, MutationTaster). It was classified as likely pathogenic by ACMG criteria. Despite repeated plasma infusions, the child experienced progressive neurological deterioration and succumbed shortly thereafter. Figure 3 illustrates the schematic representation of the ADAMTS13 protein and the location of novel variants identified in the present cases.

## Discussion

Congenital thrombotic thrombocytopenic purpura (cTTP; Upshaw-Schulman syndrome) is an ultra-rare disorder caused by severe deficiency of ADAMTS13, the metalloprotease responsible for cleaving ultra-large von Willebrand factor (UL-VWF) multimers. In the absence of ADAMTS13, platelet-rich thrombi form in the microcirculation, producing microangiopathic hemolytic anemia (MAHA), thrombocytopenia, and ischemic organ injury. Presentation is heterogeneous [1]. Neonates present with severe hyperbilirubinemia, anemia, and thrombocytopenia. Older children and adults present with episodic MAHA and thrombocytopenia, often triggered by infection, surgery, or pregnancy. Stroke, renal, and cardiac events occur even with prophylaxis.

The disease is characterized by the classical triad of thrombocytopenia, microangiopathic hemolytic anemia, and visceral ischemia. Without treatment, mortality exceeds 90%, underscoring the need for early recognition and intervention. cTTP is inherited in an autosomal recessive manner, most commonly due to biallelic pathogenic variants in ADAMTS13, either homozygous or compound heterozygous. Rarely, monoallelic mutations in ADAMTS13 in combination with clusters of single-nucleotide polymorphisms (SNPs) have been implicated, further expanding the genetic spectrum of this disorder.

Although traditionally considered extremely rare, recent analyses of large genome and exome sequencing datasets (141,456 individuals) indicate that ADAMTS13 disease alleles are considerably more common than previously recognized, with the estimated prevalence of cTTP more than 10-fold higher than reported, suggesting many patients remain undiagnosed. Over 450 ADAMTS13 variants have now been described, highlighting the mutational burden of this gene [1].

Genotype-phenotype correlations are imperfect [2]. Some trends exist: N-terminal variants and very low residual activity are associated with earlier onset. Founder variants such as c.4143_4144dupA and p.R1060W recur in certain populations. In the present article, we discuss two children with extremes of phenotype and review current literature with emphasis on pathogenicity assessment, genotype-phenotype, and treatment.

Mutational spectrum and genotype-phenotype variability in congenital TTP

Congenital thrombotic thrombocytopenic purpura (cTTP) results from biallelic pathogenic variants in ADAMTS13, the gene encoding the von Willebrand factor-cleaving protease. More than 450 variants have been reported to date, spanning all domains of the protein, including the metalloprotease, disintegrin-like, thrombospondin type 1 repeats, cysteine-rich, spacer, and CUB domains [3,4]. Missense mutations are the most common, accounting for over half of all cases. Compound heterozygosity is typical in outbred populations, whereas homozygosity is more frequent in consanguineous families. Several studies suggest that the location of the mutation influences disease severity. Variants in the N-terminal portion of ADAMTS13, particularly those within the metalloprotease, cysteine-rich, and spacer domains, often abolish enzymatic secretion or function and are associated with earlier onset and more severe clinical phenotypes [5,6]. In contrast, distal mutations affecting the thrombospondin or CUB domains may permit partial secretion or residual activity, resulting in later onset or milder disease [7]. Classic examples include the recurrent c.4143_4144dupA mutation, which has been linked to variable phenotypes ranging from neonatal onset to adult presentations even among individuals with the same genotype [8], and the common p.R1060W variant in exon 24, which demonstrates incomplete penetrance, with some homozygotes presenting only during pregnancy or with mild episodic symptoms [9].

Despite these associations, genotype-phenotype correlation remains imperfect. Data from the International Hereditary TTP Registry indicate that more than half of patients with severe biochemical deficiency present after infancy and that environmental or physiological triggers such as infection, dehydration, or pregnancy often precipitate the first clinical episode [10]. These findings highlight the role of additional modifiers, including co-inherited polymorphisms, environmental exposures, and epigenetic regulation, in shaping clinical variability.

The pathogenicity assessment of novel ADAMTS13 variants requires a multiparametric approach. In silico prediction algorithms such as SIFT and PolyPhen-2 provide initial insight into the functional impact of amino acid substitutions, while conservation analysis identifies alterations at evolutionarily preserved residues. Domain-specific scoring is relevant, since variants affecting the metalloprotease, cysteine-rich, and spacer domains are more disruptive than those in distal regions. Additional tools, such as charge-based scoring, which predicts disruption of electrostatic interactions and protein folding, and phosphorylation site prediction, which evaluates the effect of substitutions on post-translational modifications, add granularity. Codon usage bias analysis, quantified through the Relative Synonymous Codon Usage (RSCU) score, refines evaluation further by estimating translational efficiency and transcript stability. Zing et al. demonstrated that combining conservation, structural context, charge, phosphorylation, and codon usage scores improved discrimination between pathogenic and benign variants compared with any single predictor [1].

Ultimately, variant classification follows the ACMG guidelines, which integrate population frequency (absence in gnomAD), segregation with disease, computational predictions, conservation, and functional validation assays. Applying this framework, the novel homozygous missense variants identified in our patients, i.e., p.Gly316Arg in the cysteine-rich domain and p.Arg507Trp in the spacer domain, were absent from population databases, highly conserved across species, predicted deleterious by multiple in silico tools, and thus classified as likely pathogenic. In both of our cases, variants fulfilled ACMG criteria for likely pathogenicity. The p.Gly316Arg substitution in the cysteine-rich domain has not been previously reported but is absent from controls and predicted to be damaging. The p.Arg507Trp variant in the spacer domain is novel, conserved, and predicted to be deleterious, correlating with the catastrophic early-onset phenotype observed. These observations reinforce the principle that variants affecting early domains or abolishing secretion are generally associated with more severe presentations, though variability remains.

Taken together, the literature underscores that while certain ADAMTS13 mutations predispose to severe neonatal-onset disease, others permit milder or later presentations. However, the overlap between genotypes and phenotypes means that genetic testing must always be interpreted alongside biochemical assays and clinical history. Functional characterization of novel variants remains essential for clarifying pathogenicity, guiding management, and refining genotype-phenotype models.

Clinical spectrum and management of cTTP

The clinical spectrum of congenital thrombotic thrombocytopenic purpura (cTTP) in childhood is remarkably heterogeneous, ranging from catastrophic neonatal disease to episodic relapsing forms presenting in later childhood or adolescence. Neonates may manifest with severe indirect hyperbilirubinemia, anemia, and thrombocytopenia within days of birth, frequently necessitating exchange transfusion and often misdiagnosed as immune hemolysis or sepsis [1,2]. Beyond the neonatal period, children may present with recurrent episodes of microangiopathic hemolytic anemia (MAHA) and thrombocytopenia, often triggered by intercurrent infections, dehydration, or vaccinations [3,4]. Some patients follow a chronic relapsing-remitting course characterized by fluctuating cytopenias and hemolysis, while others remain asymptomatic until adolescence or early adulthood, when pregnancy or surgical stress unmasks the deficiency [5,6]. Importantly, ischemic organ involvement, including renal dysfunction, myocardial ischemia, and particularly cerebrovascular events, can occur at any stage, and strokes have been reported even in children maintained on regular plasma prophylaxis [7]. Data from the International Registry emphasize that more than half of children experience at least one acute thrombotic event, and long-term morbidity is substantial despite therapy [8]. This variability underscores the need for clinicians to suspect cTTP in any child presenting with the triad of anemia, thrombocytopenia, and schistocytosis, particularly when associated with a reactive marrow and negative Coombs test. Early-onset disease is generally associated with more severe phenotypes, reflecting near-complete loss of enzymatic activity, whereas later-onset disease may correspond to variants that allow partial activity, which is often not detectable by routine assays [1,2,9]. Data from the International Hereditary TTP Registry indicate that more than 50% of patients with severe biochemical deficiency present only after infancy, with first episodes commonly triggered by infection, surgery, or pregnancy [3]. Importantly, as noted in our cases (Figure 3), mutations localized to the N-terminal region (pre-spacer) are more frequently associated with early and severe disease, while post-spacer mutations are often linked to delayed onset and milder courses. In a large cohort, the median age of presentation for pre-spacer mutations was 24 months compared to 294 months for post-spacer mutations, underlining this distinction [4]. Furthermore, pre-spacer variants accounted for 52% of childhood presentations but only 10% of adult-onset cases [4,10]. These observations highlight the broad clinical heterogeneity of cTTP and the limitations of relying on residual activity alone as a prognostic marker.

The cases presented herein underscore the remarkable clinical heterogeneity of congenital thrombotic thrombocytopenic purpura. Despite a shared underlying mechanism of severe ADAMTS13 deficiency, the two children described exhibited strikingly divergent phenotypes. The first child manifested in later childhood with relapsing microangiopathic hemolytic anemia and thrombocytopenia, achieving long-term stability with regular plasma prophylaxis and without major neurological or renal sequelae. In contrast, the second child developed catastrophic neonatal disease characterized by severe jaundice at birth, recurrent cytopenias in infancy, and a fatal ischemic stroke before one year of age, despite supportive plasma infusions.

## Conclusions

cTTP is an ultra-rare but potentially fatal disorder. Early diagnosis with ADAMTS13 testing, genetic confirmation, and immediate plasma therapy are critical. Genotype-phenotype correlations provide broad guidance but are insufficient for individual prediction. Functional validation and registry data integration remain essential. Recombinant ADAMTS13 promises to reshape management in pediatric cTTP.
